# RNA interference in *Fasciola gigantica*: Establishing and optimization of experimental RNAi in the newly excysted juveniles of the fluke

**DOI:** 10.1371/journal.pntd.0006109

**Published:** 2017-12-12

**Authors:** Arun Anandanarayanan, Opinder Krishen Raina, Hniang Lalrinkima, Ajayta Rialch, Muthu Sankar, Anju Varghese

**Affiliations:** 1 Division of Parasitology, ICAR-Indian Veterinary Research Institute, Izatnagar, UP-India; 2 Department of Veterinary Parasitology, Dr GC Negi College of Veterinary and Animal Sciences, CSK HPKV, Palampur, HP, India; 3 Division of Temperate Animal Husbandry, ICAR-Indian Veterinary Research Institute, Mukteshwar, UK-India; 4 Department of Veterinary Parasitology, College of Veterinary and Animal Sciences, Kerala Veterinary and Animal Sciences University, Pookode, Wayanad, Kerala, India; IRNASA, CSIC, SPAIN

## Abstract

Fasciolosis caused by *Fasciola gigantica* is a neglected tropical disease but a constraint on the growth and productivity of cattle, buffaloes and sheep in the tropical countries of Asia and Africa. Resistance to commonly used anthelmintics in *Fasciola* has increased the need to search for alternative therapeutic targets. RNA interference is the current tool of choice in the search for such targets in *Fasciola*. The susceptibility of juvenile *Fasciola hepatica* to double stranded (ds) RNA induced RNAi has been established but in *F*. *gigantica* a single preliminary report on RNAi induced mRNA transcript knockdown is available. Here we optimized conditions for RNAi in the liver fluke *F*.*gigantica* targeting six genes including superoxide dismutase (SOD), σ class of glutathione-s-transferase (GST), cathepsin (Cat) L1-D, Cat B1, Cat B2 and Cat B3 that showed robust transcriptional silencing of the targets following exposure of the newly excysted juveniles (NEJs) to long (170–223 nt) dsRNA. Knockdown was shown to be concentration dependent with significant mRNA transcript suppression occurring at 5 ng / μl that showed further suppression with the increase in the dsRNA concentration. The dsRNA induced persistent silencing of the mRNA transcript of SOD and σGST up to 15 days of observation. Delivery of the long dsRNA and siRNA to the newly excysted juveniles by soaking method was found to be efficient by tracking the uptake and diffusion of Cy^3^ labelled siRNA and long dsRNA in the flukes. Off-target effects of dsRNA trigger on some of the non-target genes were detected in the present investigation on RNAi in *F*. *gigantica*. The dsRNA induced superoxide dismutase protein suppression while impact of RNAi on other target proteins was not studied. There is no *in vitro* culture system for prolonged survival of the *F*. *gigantica* and in the present study *in vitro* maintenance of the NEJs is reported for a period of 3 weeks. The present study is the first attempt on optimization of RNAi protocols in *F*. *gigantica* where long dsRNA allowed for an efficient and persistent gene silencing, opening prospects for functional validation of putative vaccine and therapeutic targets in this neglected parasite.

## Author summary

RNA interference (RNAi) is a powerful method for selectively silencing genes for the validation of potential targets for drug and vaccine development. The susceptibility of juvenile *Fasciola hepatica* to double stranded (ds) RNA induced RNAi has been established but in *F*. *gigantica* a single report of a preliminary study on knockdown of a single gene transcript exists. In the absence of other tools of reverse genetics, RNAi occupies a centre stage in the validation of gene functions in *Fasciola* species. This study focuses on *F*.*gigantica*, an economically important veterinary parasite with a zoonotic potential. Here in this study, we optimized a set of simple methods for triggering RNAi in the *F*. *gigantica* juvenile liver fluke, which shows that a robust transcriptional suppression can be readily achieved across all targets tested and with protein suppression confirmed in one of the targets. These studies also highlight the need for developing an *in vitro* maintenance system for the fluke for validation of the RNAi protocols. These findings are important for researchers aiming to employ RNAi in investigations of liver fluke biology and target validation.

## Introduction

*Fasciola gigantica* (tropical liver fluke) and *F*. *hepatica* (temperate liver fluke) are the two causative agents of fasciolosis in livestock and are economically important veterinary parasites due to the substantial production and monetary losses that these parasites cause to the livestock industry. Fasciolosis caused by *F*. *gigantica* is a neglected tropical disease but a constraint on the growth and productivity of cattle, buffaloes and sheep in the tropical countries of Asia and Africa [1, 2]. In addition to its adverse impact on livestock economy, fasciolosis is an emerging zoonosis particularly in the South American countries like Bolivia, Peru, Equador and in rural areas of central Africa and northern Asia [3, 4, 5]. In the Indian sub-continent *F*. *gigantica* is the causative agent of fasciolosis in livestock. Control measures rely mainly on anthelmintic drugs but in recent years reports of resistance to commonly used anthelmintics in *Fasciola* have emerged thereby increasing the need for alternative therapeutic targets [6, 7, 8]. The advent of new sequencing technologies facilitated the knowledge of the genomes and transcriptomes of trematodes [9, 10, 11, 12]; providing sources for seeking novel drug and vaccine targets. Whereas genome sequence information is ever expanding, studies on the genes coding for proteins of unknown function are limited. RNA interference is the method of choice for gene function analysis since classical tools of functional genomics are not available in most of these parasites [13, 14]. The complex developmental cycle, robust tegument and inability to maintain the worms for extended periods *in vitro* have slowed down the deployment of techniques involved in gene function analysis in parasitic flatworms [15, 16]. Development of RNAi-based gene silencing methods is pivotal for the effective exploitation of the increasing database resources in *Fasciola*. *Fasciola* is increasingly the focus for transcriptome and genome analyses [12, 17, 18, 19, 20, 21] but RNAi and other functional genomics tools have not been widely adopted by the liver fluke research community. In parasitic flatworms most progress has been reported for schistosomes [22, 23, 24] that has demonstrated the utility of RNAi in functional genomics in trematodes. These studies have also indicated that genes are not equally affected; off-target effects can occur and developmental stages display different susceptibility to interference [25]. Successful gene silencing by RNAi reported in *Opisthorchis viverrini*, *F*. *hepatica* and *Clonorchis sinensis* [26, 27, 28] will provide framework for utilization of this technique to investigate the function of unexplored genes that in turn might be the targets for vaccine or drug development. However, in *F*. *gigantica* research on vaccine development or drug discovery has been hindered in the absence of use of reverse genetics tools including RNAi.

Parasites express antioxidant enzymes including superoxide dismutases (SODs), glutathione-S-transferase, glutathione peroxidase, catalase and peroxiredoxins that would suppress oxidative killing by the host effector cells. Superoxide dismutase a metallo-enzyme, the main superoxide radical scavenger, protects cells from the oxidant mediated damage. The identification of the Cu/Zn-superoxide dismutase in *F*. *gigantica* [29] may suggest that antioxidative response protects the parasite against neutrophil, macrophage or dendritic cell derived reactive oxygen species but precise role of the SOD enzyme expressed in *Fasciola* species in defense against superoxide mediated killing of the parasite is not known. Glutathione-s-transferase (GST) belongs to a family of enzymes that are involved in the cellular detoxification process. The GSTs of helminths act as immune defense proteins and have significant activity with lipid peroxidation-derived carbonyls and also have the potential to neutralize exogenously derived toxins such as anthelmintics [30]. GSTs are considered a promising vaccine candidate against *Fasciola* species [27, 31]. Cysteine proteases are essential for acquiring nutrients and enabling the parasite to migrate from the intestine and through the liver [32, 33] in immunomodulation process [34, 35] and host immunoglobulin cleavage [36]. In order to understand the diverse functions performed by these classes of proteins in *F*. *gigantica*, their functional analyses using RNAi are essentially required. Therefore, we focused here on the optimization of RNAi platform in juveniles of the liver fluke *F*. *gigantica* using the above genes as targets for its establishment as a viable tool for investigating the gene function in this parasite.

## Materials and methods

### *In vitro* hatching of *F*. *gigantica* newly excysted juveniles

*Fasciola gigantica* metacercariae were harvested on polythene strips from naturally infected *Lymnaea auricularia* collected from the rural ponds and hatched into newly excysted juveniles (NEJs) *in vitro* [37]. Briefly, metacercariae were treated with 1% pepsin in 0.4% HCl prepared in sterile distilled water and incubated at 37°C for 45–60 min. The metacercariae were washed with several changes of sterile distilled water to remove the outer cyst wall debris and were further incubated for 2–3 h at 37°C in 10 ml excystment solution of 20 mM sodium dithionite (sodium hypodisulphite), 1.5% (w/v) NaHCO_3_, 0.8% (w/v) NaCl, 0.2% (w/v) taurocholic acid and 0.5% (v/v) conc. HCl in 50 ml centrifuge tube with its cap sealed firmly with parafilm (reagents used in the NEJ hatching were procured from Sigma Chemicals, USA). The cysts were subsequently washed in sterile distilled water and resuspended in serum free RPMI-1640 medium, supplemented with 50 μg /ml of gentamicin and incubated at 37°C overnight for their hatching. The NEJs that hatched from the metacercariae were filtered through a sterile nylon mesh of ~100 μm pore size in RPMI-1640 medium at 37°C for 60 min and cultured in complete RPMI-1640 medium. Species identification of the NEJs for *F*. *gigantica* was done by PCR amplification and sequencing of ITS-2 and 28S rDNA markers [38].

### *In vitro* maintenance of the NEJs

Three commercially available culture media RPMI-1640 (Hyclone, USA), DMEM (Hyclone, USA) and DME / F12 (1:1) (Hyclone, USA) were tested for supporting the *in vitro* survival and growth of the juveniles. Growth media were supplemented with the final concentrations of foetal bovine serum (10%) (Hyclone, USA) / chicken serum (50%) (Himedia, India), glucose (2%) and HEPES (25 mM) for enhancing their efficacy in extending the survival of the juveniles. Appropriate doses of antibiotics (1x streptomycin-penicillin) and amphotericin B (1 μg / ml) were added and parasite culture was maintained at 37°C in 5% CO_2_ atmosphere.

### Isolation of RNA from the NEJs and cDNA synthesis

Total RNA was extracted from the juveniles using the RNAqueous Micro kit (Ambion, USA) with the mini extraction protocol as per the manufacturer's instructions. Briefly, 100–150 NEJs treated for RNAi or as untreated controls were used for RNA isolation. Total RNA isolated with the kit was digested with DNase and quantified by Nano-drop spectrophotometer (Thermo Scientific, USA). Equal amounts of total RNA (300 ng) from all experimental groups were used for cDNA synthesis using oligo-dT primer and M-MLV reverse transcriptase enzyme (MBI Fermentas, USA). Six target genes of the fluke *F*. *gigantica* including superoxide dismutase (SOD), σ class of glutathione-s-transferase (GST), cathepsin (Cat) L1-D, Cat B1, Cat B2 and Cat B3 were selected for RNAi. The cDNA synthesized from the total RNA was subjected to PCR amplification of full length open reading frame of *F*. *gigantica* SOD (accession no: GU906887), σGST (accession no: DQ974116), cathepsin L1-D (accession no: AF239266), Cat B1 (accession no: (AY227673), Cat B2 (accession no: (AY227674) and Cat B3 (accession no: AY227673) using N and C terminal primer sequences specific to the respective genes. The PCR products representing each of the target genes, except for Cat B1 and B3, were cloned in p^DRIVE^ TA-cloning vector and the respective dsRNA trigger was generated by PCR amplification of the short sequences of these clones. However, the PCR templates for the generation of dsRNA trigger for Cat B1 and Cat B3 were amplified from the cDNA directly using a single set of primers for both the targets.

### Expression of the recombinant SOD protein

The SOD PCR product (441bp) was sub-cloned in prokaryotic expression vector pPROEXHT-b. The histidine tagged fusion protein was expressed in *Escherichia coli* BL 21 (DE3) by inducing the recombinant protein expression with 1mM IPTG at 37°C for 7–8 h. The recombinant protein was purified under denaturing conditions by Ni-NTA affinity chromatography following standard purification protocols.

### Raising of anti-SOD antibodies in rabbits

Two New Zealand white rabbits, weighing ~1kg each, were immunized with recombinant SOD protein at 100 μg (each dose) in Freund’s complete and incomplete adjuvant, respectively at 2 week intervals. Each animal received one immunization with the antigen in Freund’s complete adjuvant and two boosters with incomplete adjuvant. Rabbits were bled after 2^nd^ booster and titre of the antibodies was determined in ELISA.

### Western blotting

The newly excysted juveniles (n = ~500) from the dsRNA treated and untreated control groups, respectively were manually homogenized with a sterile micropestle in 200 μl of phosphate buffered saline (PBS), pH 7.2 in a round-bottom 2 ml microcentrifuge tube under exposure to liquid nitrogen and sonicated at 5 micron amplitude x 5 cycles of 15 sec each over ice in three biological replicates. A cocktail of protease inhibitors (1x, Sigma Aldrich) was added to each tube and the protein content in each group was quantified by Lowry method [39] and equal quantities of protein (50 μg) from dsRNA treated and untreated groups were loaded in the wells of SDS-polyacrylamide gel in each experimental repeat. The proteins were resolved in 15% SDS-PAGE using tris-glycine buffer pH 8.6 and transferred to nitrocellulose membrane in tris-glycine buffer with 20% methanol at 100 mA for 1h. The blots were washed in PBS pH 7.2 post-transfer and blocked with 5% skimmed milk in PBS for 1 h at 37°C. Membranes were probed at 37°C for 1 h in the blocking buffer containing two primary antisera (one raised against FgSOD target protein in rabbit and other raised against FgGST in rabbit as loading control normalizer) at 1:200 dilution. Following 5x5 min washes in PBS-Tween 20, membranes were reacted with goat anti-rabbit IgG-HRP conjugated secondary antibodies (Sigma Chemicals, USA) diluted 1:800 in blocking buffer. The blot was developed with 3, 3'-diaminobenzedene (1 mg / ml, W/V) (Sigma-Aldrich USA) in PBS, pH 7.2 and 30% hydrogen peroxide (1 μl / ml) as per the standard protocols. Membranes were then dried, scanned and band intensities quantified by densitometry using GelQuant.NET software provided by biochemlabsolutions.com. The relative protein quantification was done by normalization of the band intensity of the protein of interest to the intensity of the loading control band from the same sample and finally figure expressed as a percentage relative to the untreated control (100%) sample. Statistical analysis was performed using ANOVA.

### RNA interference: Design and synthesis of double stranded RNA

RNAi experiments were conducted on the newly excysted juvenile stage of the parasite. RNAi triggers used for silencing the targeted genes in the *F*. *gigantica* NEJs comprised of long double stranded (ds)RNA molecules (170–223 nt) that were generated by T7 RNA polymerase-driven transcription of single RNA strands from the target-specific PCR generated templates tailored with T7 RNA polymerase promoter sequence 5'-TAATACGACTCACTATAGGG-3'. The cDNAs coding for full length open reading frame of the SOD, σGST, Cat L1-D, Cat B1, Cat B2 and Cat B3 were PCR amplified using primer sequences specific to N- and C-termini of respective genes. These PCR products were used as template for the generation of dsRNA molecules specific to these targets. Short target sequences of SOD cDNA spanning 151–320 nucleotides (170 bp) and σGST from 57–279 nucleotides (223 bp) were PCR amplified for the generation of dsRNA triggers. The nucleotide sequence from 301–475 (175 bp) of Cat L1-D was PCR amplified for the generation of Cat L1-D dsRNA. The Cat B1 and Cat B3 dsRNAs were generated by PCR amplification of 370–572 nucleotides (203 bp) of Cat B1 and Cat B3 cDNA using a single set of forward and reverse primers designed on the short conserved sequences of these genes. Likewise, Cat B2 specific dsRNA was generated by PCR amplification of the target sequence of 351–572 nucleotides (222 bp) (S1 Fig). Each target sequence was PCR amplified using gene specific primers incorporated with T7 promoter sequence at 5' end of the sense and anti-sense primer (Table 1). *Plasmodium falciparum* knob associated histidine rich protein (PfKAHRP) gene (accession no: X92413) was used as negative control (Pfcont) as PfKAHRP gene lacked significant sequence similarity with *Fasciola* nucleotide sequence (accession nos: LN771073; LN771075; LN771081).The PfKAHRP gene fragment was PCR amplified with forward and reverse primers (Table 1) and dsRNA was generated as described for the above target genes. All the PCR amplicons were sequenced for their authenticity.

**Table 1:** Sense and anti-sense primers tailored with T7 promoter sequence at 5' end (in bold) for *in vitro* transcription of dsRNA trigger against six target genes of *F*. *gigantica* and for irrelevant control dsRNA against *Plasmodium falciparum* KAHRP gene.

Primer Name	Sequence
FgSOD–F FgSOD-R	5'-**TAATACGACTCACTATAGGG**ATATCCGCGGGACCTCATTTCA AC-3' 5'- **TAATACGACTCACTATAGGG**CCAATAACTGAGTTCACTCCGG AG-3
FgCat B1+B3-F FgCat B1+B3-R	5'- **AATACGACTCACTATAGGG**ACCGACCGTATATGCATTCATT-3' 5'- **TAATACGACTCACTATAGGG**TTGGGGAACGGGTAGGGTAAAC-3'
FgCat B-2-F FgCat B-2-R	5'- **TAATACGACTCACTATAGGG**GGCGGCAGCCAGTGCAATGAGT-3' 5'- **TAATACGACTCACTATAGGG**T TGGTAAACATCCAGGGCTGAC-3'
FgCat L1-D-F FgCat L-1 D-R	5'- **TAATACGACTCACTATAGGG**TATAAGGCGAACAAGCCCGCCG-3' 5'- **TAATACGACTCACTATAGGG**GTTGCTCAGAGAATGAAGCACT-3'
FgσGST-F FgσGST-R	5'- **TAATACGACTCACTATAGGG**AATTCGCCTTCTGCTCACTTGT-3' 5'- **TAATACGACTCACTATAGGG**GTAATACTCCTCGTCCGTTTCACC-3'
PfKAHRP-F PfKAHRP-R	5'- **TAATACGACTCACTATAGGG**GCAAAAGAAGCAAGTACTTCTAA-3' 5'- **TAATACGACTCACTATAGGG**GCAGTTCCATCTTTAGATTGTAC-3'

### *In vitro* transcription of dsRNA

*In vitro* transcription of the dsRNA trigger molecules against six target genes was carried out using commercially available *in vitro* transcription kit (TranscriptAid T7 High Yield Transcription Kit, Thermo Scientific, USA). The transcription reaction mixture was incubated at 37ºC for 2.5 h for synthesis of the dsRNA as per the instructions of the manufacturer. The dsRNA was quantified by Nano-drop spectrophotometer and analysed for the presence of discrete, correct sized product on a non-denaturing 1.5% (w/v) agarose gel. The dsRNA that gave a correct sized band and 260/280 of 1.8 was used in RNAi experiments. The purified dsRNA was stored at -80ºC for further use.

### Cy^3^-labelling of dsRNA

Out of the six dsRNAs generated against the six target genes, the SOD specific dsRNA was labelled with Cy^3^ dye. Labelling of the SOD dsRNA was carried out using SilencersiRNAi Labelling Kit (Ambion, USA) with a protocol optimized to siRNA as per the manufacturer's instructions. The GAPDH siRNA (25 nt) (commercially synthesized) was also labelled with Cy^3^ dye following the procedure used for the labelling of long dsRNA. The Cy^3^ labelled GAPDH siRNA and SOD long dsRNA were used as reporter RNA for determining the comparative efficacy of the uptake of long dsRNA and siRNA molecules by the newly excysted juveniles. The Cy^3^ labelled dsRNA and siRNA were delivered to the NEJs by soaking method and the NEJs were maintained *in vitro* for a period of 24–72 h. The flukes were washed with several changes of PBS, pH 7.2 and were observed under UV fluorescence microscope (Olympus UTV 63XC Fluorescent Microscope, Japan) over a period of 24 to 72 h for the incorporation of the long dsRNA and siRNA in the fluke tissue.

### Delivery of dsRNA to NEJs

Delivery of the RNAi trigger molecules to newly excysted juveniles was attempted by soaking and electroporation to determine the efficacy of each method in the delivery of dsRNA molecules to the parasite tissue.

### Soaking method

*F*. *gigantica* NEJs were soaked in solutions of long dsRNA molecules dissolved to defined concentrations of 5 ng / μl to 150 ng / μl in RPMI-1640 in 24 well culture plates. Soaks were handled in each well alongside the untreated (with no dsRNA) controls and each assay was carried out in three replicates. The NEJs were initially exposed to a defined concentration of dsRNA for 24 h in 1 ml RPMI-1640 medium that was diluted to 2 ml with fresh RPMI-1640 medium for next 48 h, thus allowing for the exposure of the NEJs to dsRNA for a period of 72 h. However, for studies on persistence of RNAi in the fluke, the NEJs were exposed to dsRNA in RPMI-1640 medium for 48 h and the medium was replaced with fresh RPMI-1640 medium without dsRNA. Thereafter, the juveniles were maintained in this medium for the next 13 days of study without dsRNA, with change of medium every 48 h. For each experiment ~150 NEJs were used per soak. The NEJs were maintained aseptically in RPMI-1640 at 37ºC in 5% CO_2_ atmosphere. Worms were visually assessed for aberrant motility or morphological changes during the culture period. At the end of each soak experiment, worms were processed for total RNA extraction.

### Electroporation

The dsRNA molecules were also delivered to the NEJs by electroporation. Square wave electroporation was carried out in BTX Electro Square Porator (BTX Square Wave Electroporation system ECM830 BTX, USA). The flukes were electroporated with different concentrations of dsRNA. The NEJs were maintained in RMI-1640 medium at 37ºC in 5% CO_2_ incubator for 24 h post-electroporation for measuring the mRNA transcript knockdown.

### Quantitative real-time PCR for estimation of the mRNA transcript suppression

Five sets of primers were synthesized for quantitative real-time PCR (qRT-PCR) amplification of six target genes post-RNAi treatment (Table 2). Primers specific to *F*. *hepatica* GAPDH (accession no: AY005475) were used for the amplification of the GAPDH sequence in *F*. *gigantica* due to high identity between the relevant orthologues. Real-time PCR was carried out in Applied Bio Systems 7500 v 2.3 Stepone plus and Applied Bio Systems 7500 v 2.0.6 fast Real-time PCR System. The primers for real-time PCR analysis were designed outside of the target sequences of the dsRNA trigger to avoid non-specific amplification of reverse transcribed dsRNA. Reactions were performed in triplicate with initial incubation of the reaction mixture at 50°C for 2 min, followed by 15 min exposure at 94°C and cycling conditions of 94°C, 15 sec; 60 ^o^C, 30 sec and 72°C, 30 sec (40 cycles) using Maxima SYBR Green / ROX qPCR Master Mix Kit (Thermo Scientific, USA). Fluorescence was detected during the extension step and melt curve analysis was performed after PCR cycling for the presence of a single peak for each expected amplicon. The relative transcript levels were analyzed by the 2^−ΔΔCt^ method [40] using *F*. *gigantica* GAPDH as the internal reference gene for normalization. The differences of Ct for the target and reference genes were calculated (ΔCt = Ct (target gene)–Ct (endogenous control) for each condition and normalized by subtracting the values obtained for treated and non-treated samples (ΔΔCt = ΔCt (dsRNA treated worms)– ΔCt (untreated control worms). Results are presented as the mean ± standard error (SE) of the unit value of 2^−ΔΔCt^ from three independent experiments. Statistical analysis was performed by paired Student’s t-test on ΔCt values, with SPSS 20 (IBM) software; with P values of ≤0.05 considered significant. Experiments were repeated ≥3 times. Significant differences between dsRNA treated and untreated control worms are indicated (*, P<0.05; **, P<0.01; ***, P<0.001).

**Table 2:** Primers designed for qRT-PCR estimation of the mRNA transcript knockdown of *F*. *gigantica* target genes post-RNAi.

Primer Name	Sequence
FgSOD -F FgSOD-R	5'-TGGAGATACAACGAATGGTTGT-3' 5'- CCAATAACTGAGTTCACTCCGG-3'
FgCat B1+B3-F FgCat B1+B3-R	5'-TCCAGTTGTAGTTCGTGTTGG-3' 5'- TTGGGGAACGGGTAGGGTAAAC-3
FgCat B-2-F FgCat B-2-R	5'- GTTGTGGTTCCTGTTGGGCCAC-3' 5'- AACCGGTTCGATTTTCCCAAGT-3'
FgCat L1-D-F FgCat L-1 D-R	5'- GTTACTCTCACGCGGTATCCCG-3' 5'- AGCTCTTTCGTTCTTCCTAAAC-3'
FgσGST-F FgσGST-R	5'- ATTCCGTGGGCGAGCAGAACC-3' 5'- GTAATACTCCTCGTCCGTTTCACC-3'
FgGAPDH-F FgGAPDH-R	5'- GGCTGTGGGCAAAGTCATTC-3' 5'-AGATCCACGACGGAAACATCA-3'

## Results

### *In vitro* maintenance of *F*. *gigantica* NEJs

**Fig 1:**
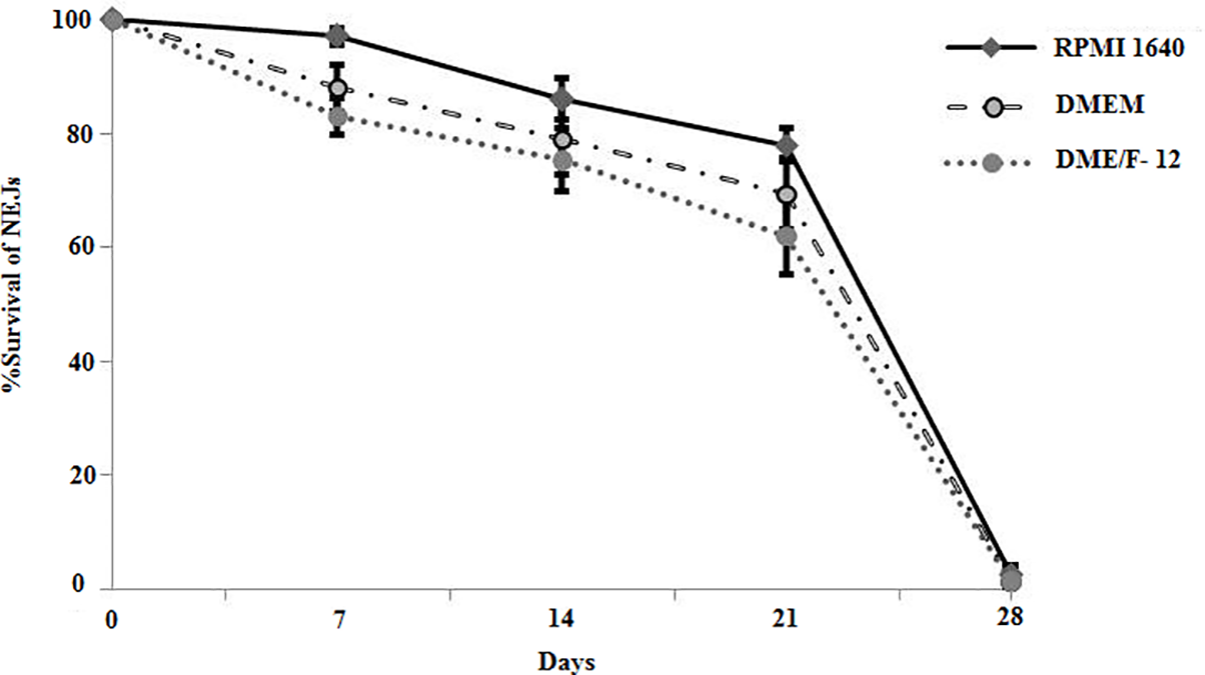
Survival of *F*. *gigantica* NEJs in RPMI-1640, DMEM and DME/F-12 culture media supplemented with 10% foetal bovine serum, 2% glucose and 25 mM HEPES. NEJs showed higher % survival (≥ 80%) in RPMI-1640 which was lower in DMEM and DME/F-12 medium (≥75% and ≥72%), respectively at 3 weeks of culture. All flukes died by 28–30 days of culture.

Three commercially available culture media including RPMI-1640, DMEM and DME/F12 (1:1) supplemented with varying concentrations of foetal bovine serum (5–10.0%), glucose (1–3.0%) and HEPES (5 mM to 25 mM) were tested for their ability to sustain a prolonged culture of the fluke NEJs. Of the three culture media tested RPMI-1640 showed extended survival of the parasite. RPMI-1640 supplemented with 25mM HEPES supported survival and growth of the NEJs for more number of days and resisted the pH change of the culture medium. The NEJs (≥80.0%) survived for three weeks in RPMI-1640 medium supplemented with 10% foetal bovine serum and 2% glucose with 25mM HEPES. However, in the 4^th^ week of culture a rapid decline in the viability of the flukes was detected and all the flukes died by 28–30 days of culture (Fig 1). Interestingly, in three experiments ≤ 40% the juveniles survived up to 6 weeks of *in vitro* culture with no morphological changes in the tegument. The flukes survived for 10–12 days of culture in the RPMI-1640 medium supplemented with 25% and 50% concentrations of chicken serum, respectively. The worm viability was assessed as a visual measure of worm motility and morphology; non-motile worms with a visually disrupted tegument considered dead.

### Delivery of RNAi molecules to NEJs

Target specific dsRNA was *in vitro* transcribed from each of the target PCR template and delivered as RNAi trigger for silencing of specific genes in the NEJs of the fluke either by soaking or by electroporation. Square wave electroporation was carried out in the NEJs (n = 100) at 125V, 30 milli-seconds in 2 mm gap cuvettes in 100 μl of RPMI-1640 with SOD specific dsRNA at 5 and 10 ng / μl concentration, respectively. The control group of NEJs received the same pulse rate but no dsRNA. Another group of NEJs (n = 100) was exposed to the same concentrations of dsRNA as a soak control in the soaking method. The results showed a mean knockdown of the mRNA transcript of 71.0% at 5 ng /μl dsRNA and 77.0% at 10 ng /μl dsRNA concentration in the electroporated groups of NEJs that was comparable to the mean mRNA transcript knockdown of 67.0% and 71.0%, respectively in the NEJs treated with dsRNA by soaking method (Fig not given). Soaking method being simple and less technically demanding was used as a standard protocol for dsRNA delivery to silence a range of virulence gene targets.

**Fig 2:**
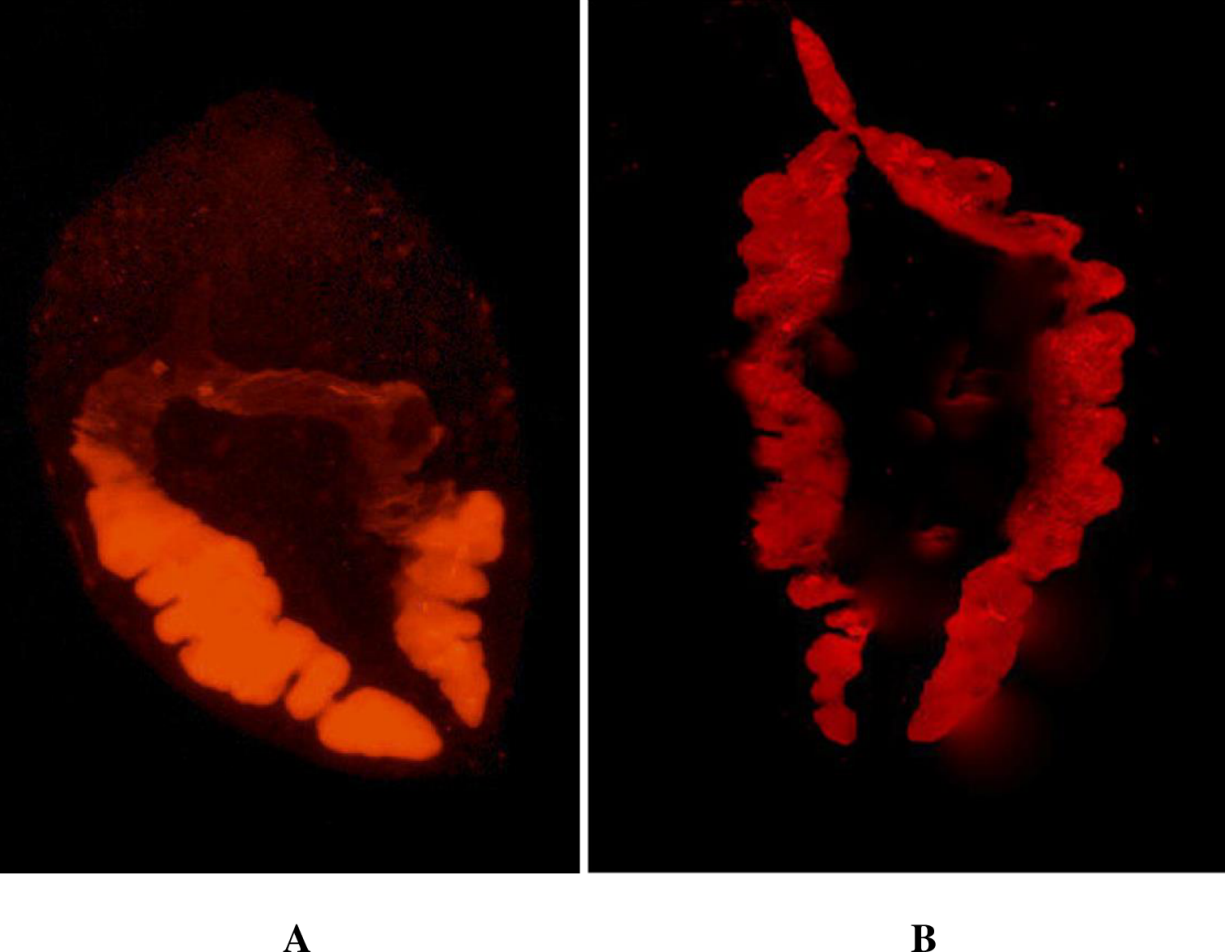
UV fluorescence microscopy of *F*. *gigantica* NEJs exposed to Cy^3^ labelled long dsRNA and siRNA by soaking method. Figure shows uptake of long dsRNA (A) and siRNA (B) through the gut of the parasite and both long and short dsRNA are widely diffused in parenchyma at 72 h (100 x magnifications).

The comparative efficacy of the uptake of long dsRNA and siRNA by the NEJs was analyzed under UV fluorescence microscope using Cy^3^ labelled long dsRNA and siRNA molecules. The exposure of the NEJs to SOD specific Cy^3^ labelled long dsRNA (210 nt) and Cy^3^ labelled GAPDH siRNA (25 nt) in two respective groups indicated that uptake of both long dsRNA and siRNA was through the gut of the fluke that diffused through the adjacent parenchyma of the NEJs. The Cy^3^ labelled dsRNA and siRNA diffused in the parenchyma adjacent to the gut in 24 to 72 h. There seemed no difference in the efficacy of the uptake and diffusion of long dsRNA and siRNA molecules in the tissues of the fluke as visualized by Cy^3^ dye fluorescence in the microscope (Fig 2). Thereafter, all experiments on RNAi were performed with long dsRNA only.

### Long dsRNA induces silencing of the target genes and display concentration dependent impacts on target transcript abundance

#### Silencing of SOD mRNA transcript

**Fig 3:**
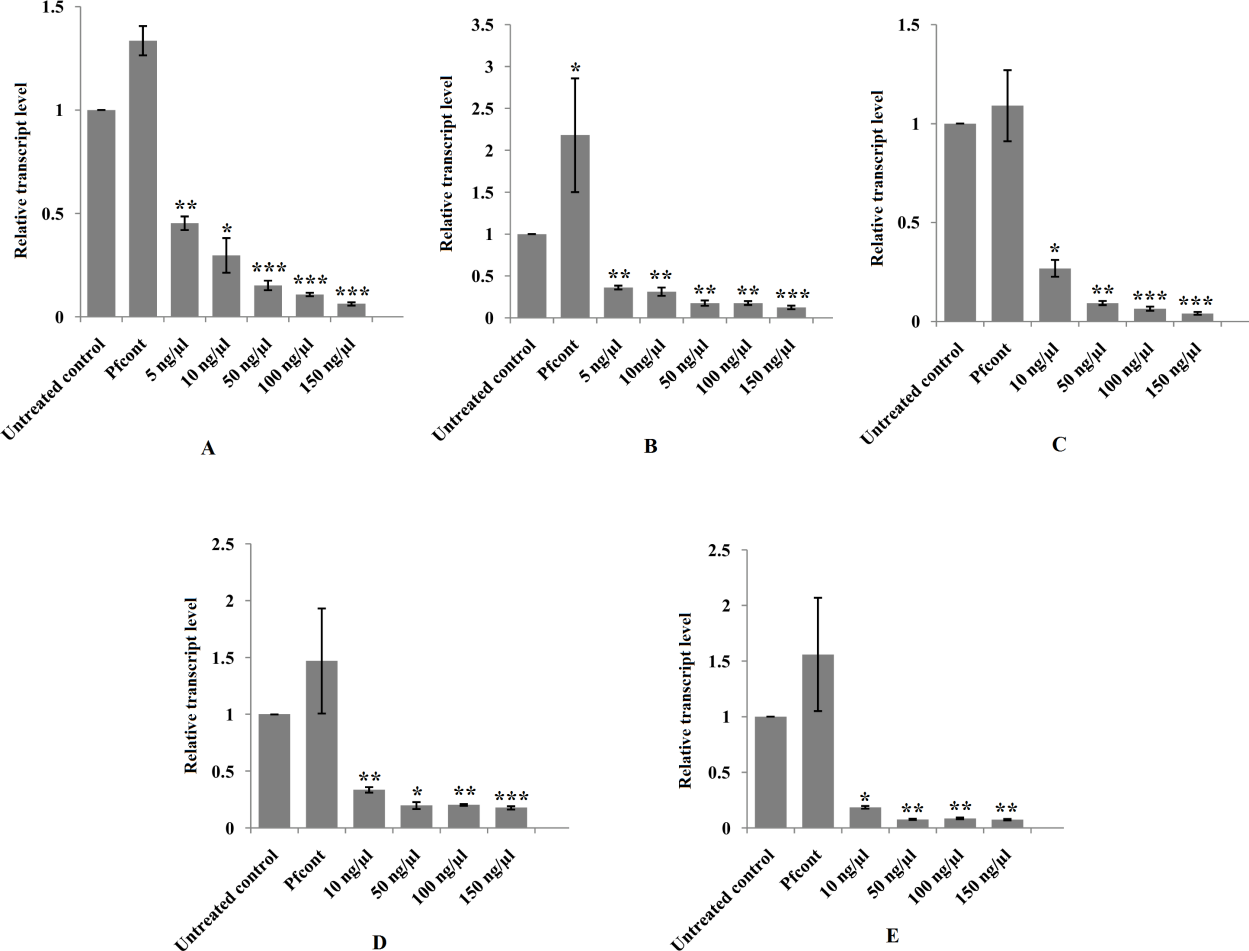
Relative transcript levels of SOD (A), σGST (B), CatL1-D (C), Cat B1-B3 (D) and Cat B2 (E) genes showing dsRNA concentration dependent suppression of the mRNA transcript at 72 h post-RNAi. Results are presented as the mean ± standard error (SE) of the unit value of 2^−ΔΔCt^ from three independent experiments. P values of ≤0.05 were considered significant. Experiments were repeated ≥3 times. Significant differences between dsRNA treated and untreated control worms are indicated (*, P<0.05; **, P<0.01; ***, P<0.001). (Pfcont = *P*. *falciparum* negative control).

The NEJs were exposed to different concentrations of SOD specific dsRNA by soaking the juveniles in the dsRNA containing RPMI-1640 medium. The newly excysted juveniles were exposed to concentrations of 5 ng / μl, 10 ng / μl, 50 ng / μl, 100 ng / μl and 150 ng / μl, respectively of dsRNA for time periods of 24 to 72 h in five groups of 150 NEJs / group and the silencing effect of the dsRNA on the target gene studied at transcriptional level by qRT-PCR showed reduction of mRNA transcript was dsRNA concentration dependent. The mean mRNA transcript suppression achieved at 5 ng / μl dsRNA treatment was 55.0% (0.45±0.03, P<0.01) while at 50 ng / μl dsRNA concentration the mRNA transcript suppression of 85.0% (0.15±0.02, P<0.001) was significantly higher. However, further increase in the dsRNA concentration from 50 ng / μl to 150 ng / μl produced no significant difference (P>0.05) in the mRNA transcript knockdown (Fig 3A). Results of these studies indicated that there was a rapid and robust mRNA reduction at 24 h and the silencing effect persisted for 72 h of study.

#### Silencing of σGST mRNA transcript

The NEJs (n = 150) were exposed to σGST dsRNA trigger at the concentrations of 5 ng / μl, 10 ng / μl, 50 ng / μl, 100 ng / μl and 150 ng / μl RPMI medium, respectively for a period of 24–72 h in five groups. The qRT-PCR results showed silencing effect at 24 h of dsRNA exposure persisting for 72 h of observation. The mean mRNA transcript knockdown at 5 ng / μl of dsRNA was 64.0% (0.36±0.02, P<0.01) that was closer to the suppression of 69.0% (0.31±0.05, P≤0.01) achieved at 10 ng / μl dsRNA. However, there was a significant difference in the suppression of the target mRNA at higher concentration of 50 ng / μl dsRNA trigger with mean suppression of 82.0% (0.18±0.03, P<0.01) that did not significantly decrease (P> 0.05) further with the increase in the dsRNA concentration from 50 ng / μl to 150 ng / μl (Fig 3B).

#### Silencing of cathepsin mRNA transcript

Newly excysted juveniles (n = 150) were also exposed to Cat L1-D, Cat B1, Cat B 2 and Cat B 3 dsRNA triggers for a period of 24–72 h at their varying concentrations. The NEJs were soaked in Cat L1-D dsRNA molecules in RPMI medium at 10 ng / μl, 50 ng / μl, 100 ng / μl and 150 ng / μl concentrations, respectively. Mean suppression of the mRNA transcript of 73.0% (0.27±0.04, P<0.05) was observed at 10 ng / μl of the dsRNA trigger and with the increase in the dsRNA concentration to 50 ng / μl a significant suppression in the transcript level of Cat L1-D to 91.0% (0.09±0.01, P<0.01) was achieved (Fig 3C). However, no further decrease in the transcript level was observed with the increase in dsRNA concentration to 100 ng / μl and 150 ng / μl, respectively (P>0.05).

Likewise, the NEJs were exposed to Cat B (B1 and B3) dsRNA molecules at 10 ng / μl, 50 ng / μl, 100 ng / μl and 150 ng / μl concentrations in RPMI culture medium. The silencing effect measured by qRT-PCR from 24 to 72 h of dsRNA exposure of the parasite indicated mRNA transcript suppression of 66.0% (0.34±0.02, P <0.01) at 10 ng / μl and 80.0% (0.20±0.03, P <0.05) at 50 ng / μl. However, with further increase in the dsRNA concentration from 100 ng / μl to 150 ng / μl no significant difference (P>0.05) in the suppression of the mRNA transcript was detected (Fig 3D).

Analysis of the suppression of Cat B2 mRNA transcript at 10 ng / μl, 50 ng / μl, 100 ng / μl and 150 ng/μl concentrations of dsRNA showed significant mRNA transcript knockdown of 82.0%, (0.18±0.01, P<0.05) at 10 ng / μl and further suppression of mRNA transcript to 92.0% (0.08±0.005, P<0.01) at 50 ng / μl. However, with further increase in the dsRNA concentration from 100 ng / μl to 150 ng / μl no significant difference (P>0.05) in the suppression of the mRNA transcript was detected (Fig 3E).

#### Long dsRNA transcript knockdown specificity

*Plasmodium falciparum* PfKAHRP dsRNA was used at a single concentration of 50 ng / μl of medium as an irrelevant (negative) control for determining the off-target effects of the control dsRNA trigger. PfKAHRP dsRNA caused statistically significant over-expression 118.0% (2.18±0.67, P<0.05) of the σGST mRNA transcript (Fig 3B). However, the relative transcript levels of other target genes were not affected significantly (P>0.05) by exposure of the NEJs to this dsRNA trigger at the given concentration (Fig 3A, 3C, 3D and 3E).

**Fig 4:**
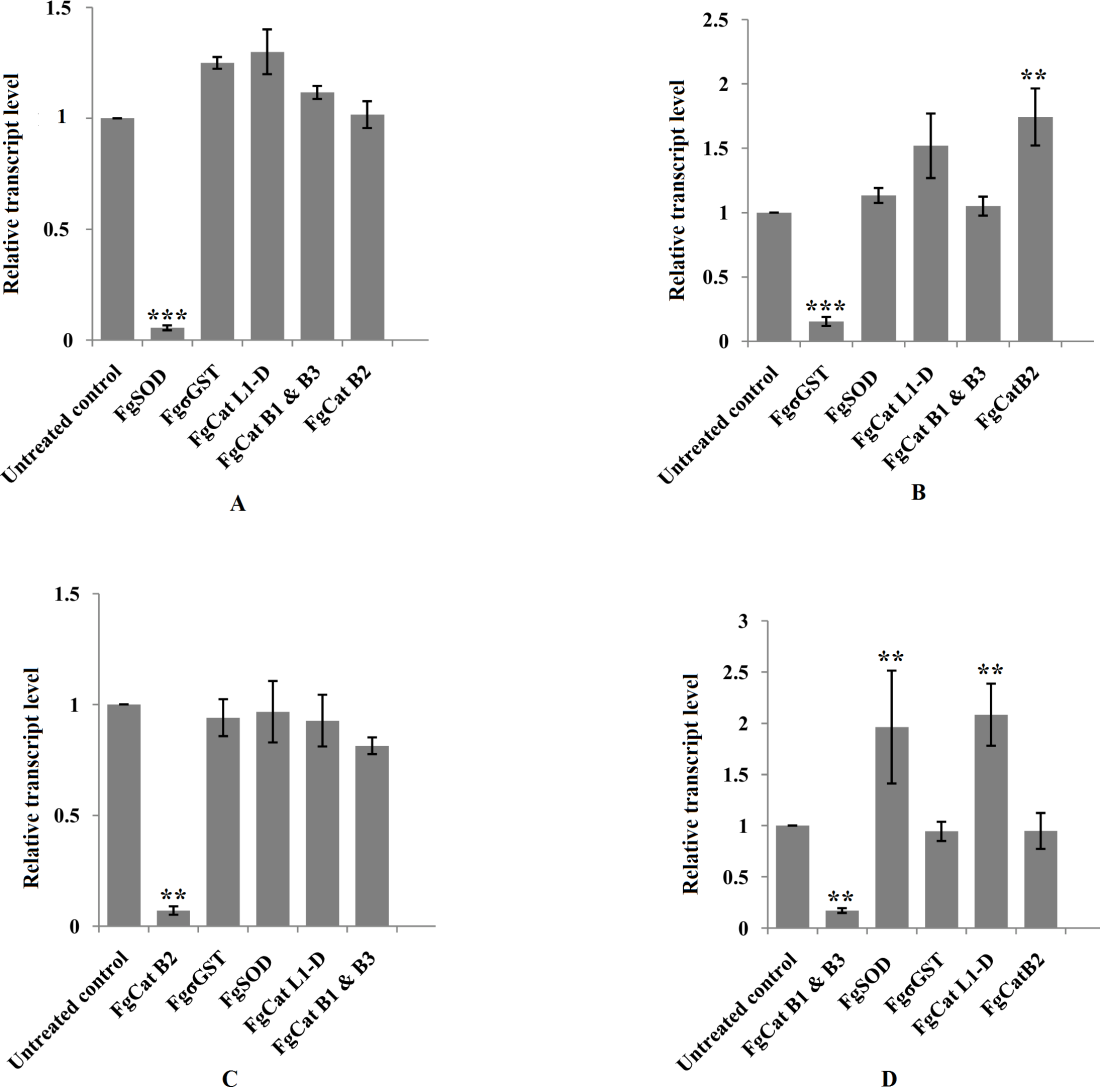
Off-target effects of the treatment of *F*. *gigantica* NEJs with SOD (A), σGST (B), Cat B2 (C) and Cat B1-B3 (D) specific dsRNA trigger on non-targeted mRNA transcripts at 72 h post-RNAi. Results are presented as the mean ± standard error (SE) of the unit value of 2^−ΔΔCt^ from three independent experiments. P values of ≤0.05 were considered significant. Experiments were repeated ≥3 times. Significant differences between dsRNA treated and untreated control worms are indicated (*, P<0.05; **, P<0.01; ***, P<0.001).

Off-target effects of gene specific dsRNAs used at a single concentration of 50 ng / μl on the other non-target genes were also analyzed. Exposure of the NEJs to SOD specific dsRNA (50 ng / μl) did not alter the expression of σGST, CatL1-D, Cat B1, Cat B2 and Cat B3 mRNA transcripts (Fig 4A). Likewise, σGST dsRNA (50 ng / μl) trigger did not influence the expression of the targets like SOD, CatL1-D, Cat B1 and Cat B3 in terms of off-target knockdown or up-regulation of transcripts but for Cat B2 where a significant up-regulation of mRNA transcript to 74% (1.74±0.22; P<0.01) was observed (Fig 4B). The off-target effects of Cat B2 dsRNA (50 ng /μl) on the expression of SOD, σGST, Cat L1-D, Cat B1 and Cat B3 were statistically insignificant (P>0.05) (Fig 4C). However, significant up-regulation of the SOD 96.0% (1.96 ±0.55 P<0.01) and Cat L1-D 108% (2.08±0.30 P<0.01) mRNA transcripts were observed in the groups of NEJs exposed to Cat B1 & Cat B3 dsRNA at 50 ng / μl concentration (Fig 4D). The off-target effects of the Cat L1-D specific dsRNA on other non-target genes was not analyzed in this study.

#### Long dsRNA induces persistent gene silencing

**Fig 5:**
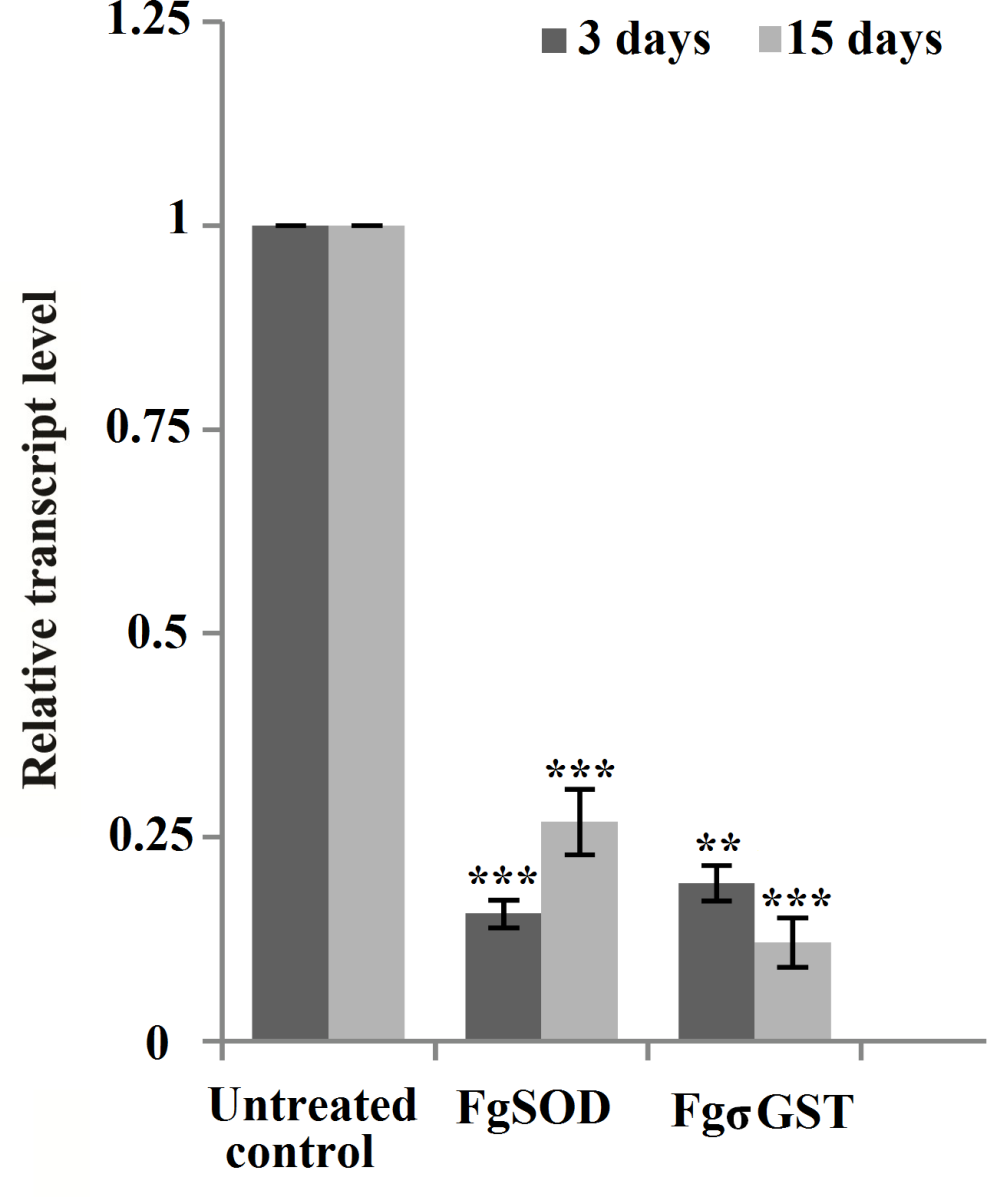
Persistence of mRNA transcript suppression at 15 days post-dsRNA treatment. The *F*. *gigantica* NEJs treated with 50 ng / μl dsRNA specific to SOD and σGST genes analyzed at 3 and 15 days post-RNAi, respectively showing significant knockdown of the mRNA transcripts post-3^rd^ and 15^th^ day of exposure.

Persistence of the gene silencing was studied in two target genes *viz*. SOD and σGST at 15 days of *in vitro* culture. The mRNA transcript knockdown measured in both the groups indicated there was a significant suppression of the SOD mRNA transcript to 73.0% (0.27±0.04, P<0.001) and for σGST transcript suppression achieved was 88.0% (0.12±0.03, P<0.001) (Fig 5).

Triggering highly significant mean transcript knockdown at 3 and 15 days, respectively of the above six targets did not impact on the survival (97% and 85% at 3 and 15 days post-exposure) or on the behavior of juveniles maintained *in vitro*, as determined by visual observations.

### Transcript knockdown leads to suppression of SOD target protein

**Fig 6:**
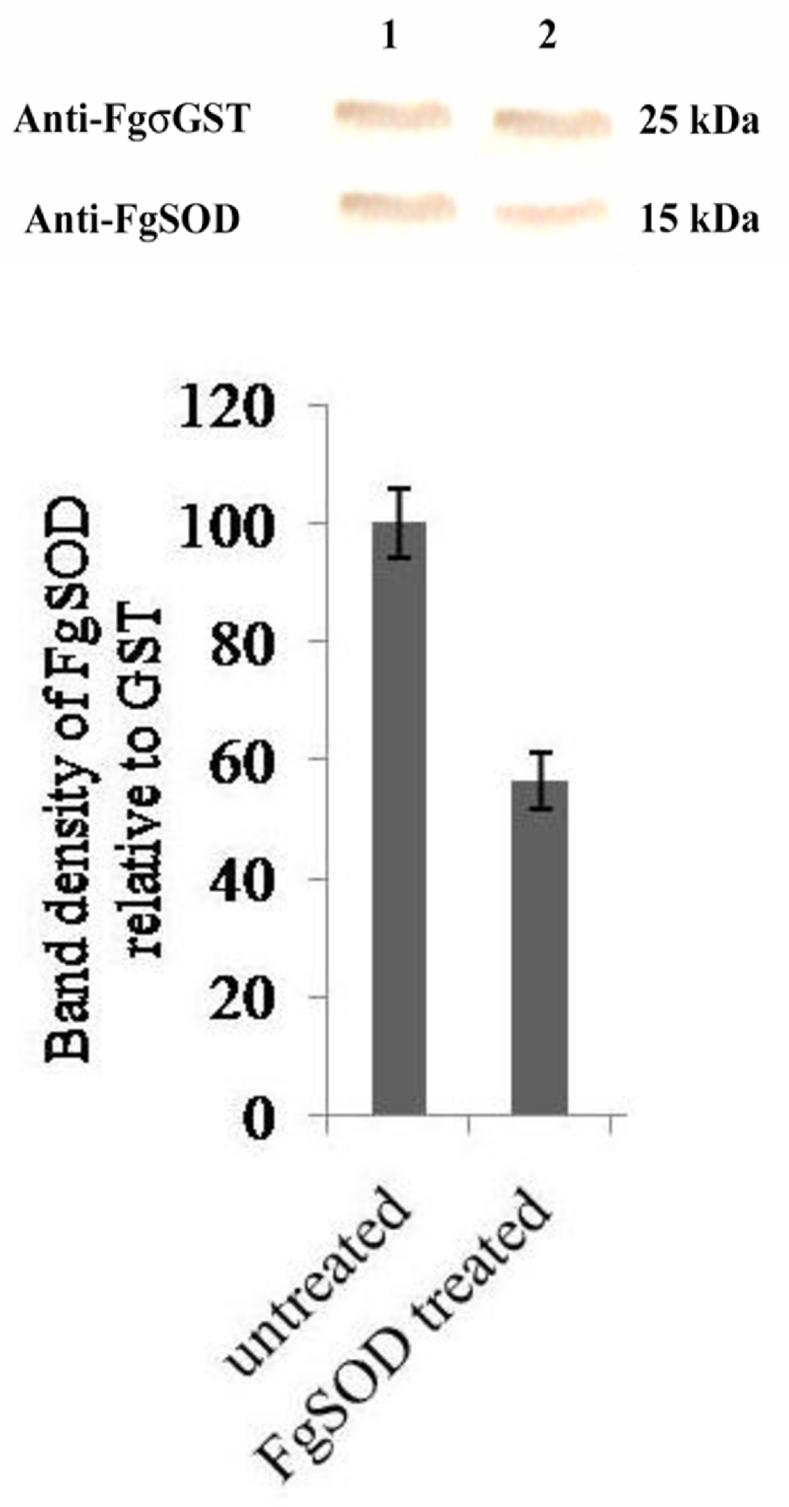
Western blot analysis of *F*.*gigantica* SOD protein suppression by SOD specific dsRNA treatment. Densitometry analysis of protein bands generated by immunoblot of NEJs crude protein extract at 9 days post-exposure to SOD specific long dsRNA. Upper panel: σGST antiserum probed bands (25kDa) in untreated (1) and dsRNA treated flukes (2). Lower panel: SOD antiserum probed bands (15kDa) in untreated (1) and dsRNA treated flukes (2). Bar graphs illustrate densitometric analyses of western blot protein bands, where target band density is normalized to loading control band density and expressed relative to untreated sample (where untreated control = 100%). Statistical significance is indicated relative to the effects of untreated control. Experiments were repeated 3 times. Significant differences between dsRNA treated and untreated control worms are indicated (*, P<0.05; **, P<0.01; ***, P<0.001). Symbols represent mean ±SEM.

Protein suppression after the robust mRNA transcript knockdown of the above target genes by RNAi was studied in SOD protein only. The NEJs were exposed to the concentration of 50 ng / μl of the SOD specific dsRNA for an initial period of 72 h and *in vitro* maintained in RPMI-1640 medium without dsRNA for a further period of 6 days before being analyzed for the protein suppression. Western blot carried out with anti-FgSOD antibodies and subsequent densitometric analysis detected significant suppression of the SOD protein (56.4 ±5.8, P<0.05, n = 3) in the group of NEJs treated with SOD specific dsRNA (Fig 6). Suppression of other target proteins on account of RNAi was not studied in the present work.

## Discussion

This study aimed at developing a standardized set of RNAi protocols applicable to *F*. *gigantica* NEJ as a means of using this robust technique in gene silencing. Recent studies on *F*. *hepatica* have shown that transcriptional knockdown was triggered in several virulence gene targets simply by soaking NEJs in a solution of tissue culture media containing long dsRNA or siRNA trigger molecules [41, 42, 43, 44]. The authors carried out in-depth optimization of variables thereby showing that transcript knockdown was concentration dependent that was invariably rapid and persistent. *In vitro* soaking method represented a relatively simple means to achieve targeted transcript and protein knockdowns in the absence of advanced manipulations such as electroporation, transfection or genetic transformation [45, 46, 47]. In the present study on optimization of the dsRNA delivery to the selected targets of *F*. *gigantica* SOD, σGST Cat L-1 D, Cat B1, Cat B2 and Cat B3 the soaking of the NEJs in the dsRNA containing culture medium was an efficient method. The uptake of the dsRNA trigger by the parasite was visualized directly by Cy^3^-labelled dsRNA and siRNA. The Cy^3^-labelled dsRNA and siRNA were incorporated in the gut of the juvenile flukes as early as 24 h of exposure. Not only was the uptake of these RNA molecules through the gut route quick but widespread diffusion of the RNA trigger in the fluke parenchyma was seen at 72 h of exposure of the NEJs. Also, delivery of the dsRNA molecules to the parasite attempted by simple soaking and electroporation respectively revealed soaking method equally efficient to electroporation; prompting us to carry out all experiments with simple soaking as the method of dsRNA delivery. The uptake of the RNAi molecules by soaking method will be suited for the targets in the gut and its adjoining areas in the fluke parenchyma but for the targets of the tegument or sub-tegumental tissues alternative approaches like electroporation, delivery by polymers, viral and non-viral vehicles etc may be more efficient.

The mRNA transcript knockdown was observed in all of the six virulence gene targets in the present study. We investigated concentrations from 5 ng / μl to 150 ng / μl of SOD dsRNA for their specific silencing effects. The reduction in the transcript level was concentration dependent with optimum suppression observed at 50 ng / μl concentration. Likewise, using variable concentrations of dsRNAs for hitting other targets like σGST, Cat L1-D, Cat B1, Cat B2 and CatB3 showed the knockdown effect was dose dependent with increasing concentrations of the trigger molecules producing enhanced target mRNA transcript knockdown. However, the off-target effects studied with five of our dsRNA triggers showed non-specific effects in terms of up-regulation of mRNA transcripts of Cat B 2 on exposure of the NEJs to σGST dsRNA trigger and with Cat B1 & B3 dsRNA trigger up-regulation of SOD and Cat L1-D mRNA transcripts occurred. These off-target effects on some of the unrelated genes could have happened due to the use of single and higher concentration (50 ng /μl) of each dsRNA trigger in these experiments. However, unfortunately, we did not study the off-target effects of these dsRNA triggers at their lower concentrations. The off-target effects have been reported earlier in other trematodes [48, 49] and indicate difficulties in the optimization of the RNAi protocols in these parasites.

Likewise, treatment of the NEJs with PfKAHRP negative control dsRNA (50 ng /μl) lead to significant (P<0.01) up-regulation of the σGST mRNA transcript. This up-regulation was observed in the treatment groups of CatB1 and CatB3 also but was not statistically significant (P>0.05). The up-regulation of σGST gene caused by this negative control could not be explained, though it seems likely that the control dsRNA used at a higher concentration caused the off-target effect. This up-regulation phenomenon has been reported in other parasites also including *Schistosoma* spp where increased levels of target genes were observed on treatment with an irrelevant control siRNA [48, 49, 50]. Whether this represents a generalized non-specific effect related to the presence of any dsRNA needs further investigation.

Persistence of the transcript knockdown of SOD and σGST targets was observed for 15 days of the study. This persistence of the silencing effect shows the potential of the RNAi in gene silencing for drug and vaccine target validation. But, all these gene manipulations did not trigger any measurable changes in NEJ viability or on their behaviour *in vitro*. Suppression of the SOD protein due to RNAi was achieved to a significant level but protein suppression in the other targets could not be investigated due to the paucity of the metacercariae.

Despite the successful knockdowns, describing extremely rapid RNAi dynamics in the NEJs of two isolates of *F*. *hepatica* [33], the study contrasted in other strain of *F*. *hepatica* where there was a longer lag phase for protein suppression in the RNAi [27]. These findings indicate that there could be a possibility of liver fluke isolate-specific differences in RNAi mechanisms and evidence for variable lag periods between transcript and protein suppression; the latter highlighting the need for careful assessment of target dynamics prior to the application and interpretation of phenotypic assays. Variable RNAi susceptibility has been observed between strains of the nematode *Caenorhabditis elegans* [50] where inter-strain differences in RNAi competency correlate with differences in the capacity to mount an anti-viral response. But in the present study on gene silencing in *F*. *gigantica* a single wild strain of the parasite, collected from the naturally infected *Lymnaea auricularia* from a single location, was used. Therefore, no comparative evaluation on the inter-strain differences on the RNAi competency in *F*. *gigantica* was carried out in the present investigation.

The transcript knockdown in *F*. *hepatica* persisting for several days following exposure to dsRNA suggests the presence of an RNA dependent RNA polymerase like secondary siRNA-based amplification system [27, 41]. The persistent knockdowns achieved in the studies by several authors suggest the amenability of *F*. *hepatica* NEJ to RNAi. In the present study on *F*. *gigantica* persistent silencing of mRNA transcript was also observed. There are reports of persistent silencing effects in schistosomes where RNAi lasts upto 40 days and in *O*. *viverrini* for at least nine days [26]. In *C*. *elegans* RNA dependent RNA polymerase is responsible for the amplification of the silencing signal [50]. However, the mechanisms responsible for the transitive RNAi effect have not been worked out in *Fasciola* species and warrants further investigation.

Recent advances in transcriptomic, genomic and functional-genomic resources have enhanced our ability to probe the fundamental biology of and identify and validate therapeutic targets in parasitic helminths. The currently available resources of draft genome [17] and several developmentally-staged transcriptomes [11, 12] in *Fasciola* species needs to be exploited by molecular tools like RNAi. Similarly, advanced proteomics and sub-proteomic methods provide tools for advancing our understanding of fluke virulence and the host-parasite interface [51]. However, the effective use of these tools has partly been hindered by the absence of an effective *in vitro* maintenance system for *Fasciola* juveniles and adult stages. In contrast, schistosomes can be maintained quite simply *in vitro* for many months in serum-supplemented culture medium, a method that has supported several functional genomics studies in schistosomes [45, 52], while similarly simple methods have supported RNAi studies in *Opisthorchis viverrini* [26] and *Clonorchis sinensis* [28]. Earlier studies using different culture media supplemented with bovine, human or chicken sera did not give consistent results on prolonged culture of *F*. *hepatica* [53, 54]. However, recently McCusker *et al*. [55] reported a new set of methods for maintaining *F*. *hepatica* juveniles *in vitro* that enabled to keep fluke alive *in vitro* for at least 6 months, as well as stimulating the development of characteristics that resemble adult parasites. These developments on the *in vitro* culture of *F*. *hepatica* will support studies on functional analyses of unknown genes; enabling the validation of new drug and vaccine targets. But, in *F*. *gigantica* still no *in vitro* culture method has been developed for newly excysted juveniles and adults that will support the growth and survival of the fluke for a prolonged time. In the present study *F*. *gigantica* NEJs could be maintained for a period of 3 weeks only in RPMI-1640 supplemented with 10% foetal bovine serum, 2% glucose at 25 mM HEPES. Report of chicken serum enabling to keep *F*. *hepatica* alive for 6 months *in vitro* [55] prompted us to supplement RPMI-1640 medium with 25% and 50% chicken serum but it did not change the survival rate of the *F*.*gigantica* NEJs in the present report and this needs further studies with other culture conditions. The lack of prolonged *in vitro* culture methods in *F*. *gigantica* will hinder studies on the RNAi suppression of such targets with long lag phase between the transcript knockdown and protein suppression as has been reported in *F*. *hepatica* [27], thus highlighting the urgent need for development of an *in vitro* culture system in *F*.*gigantica* for optimization of RNAi protocols and exploitation of the genes for vaccine and drug target validation.

### Conclusions

The current study has optimized the RNAi protocols in *F*. *gigantica* where long dsRNA was delivered to the NEJs by simple soaking method that allowed for an efficient and persistent gene silencing up to 15 days of observation, opening prospects for functional validation of putative vaccine and therapeutic targets. However, off-target effects of the dsRNA induced silencing of the specific genes is a concern for *in vivo* studies on RNAi in the parasite. Also, silencing of the proteins coded by the genes targeted in the present study for their phenotypic effects in the parasite needs to be studied.

## Supporting information

S1 Fig Schematic representation of the positions of primers used for PCR amplification of the target regions of *F*. *gigantica* SOD, σGST, Cat L1-D, Cat B1 & B3 and Cat B2 cDNAs for the generation of dsRNA specific to each gene (Abbreviation; ORF = Open reading frame). (TIF)
