# Supplementary figures and images for: RNA interference in Fasciola gigantica: Establishing and optimization of experimental RNAi in the newly excysted juveniles of the fluke
Source: PLoS Negl Trop Dis. 2017 Dec 12;11(12):e0006109. doi: 10.1371/journal.pntd.0006109 (PMC5749881; doi:10.1371/journal.pntd.0006109)

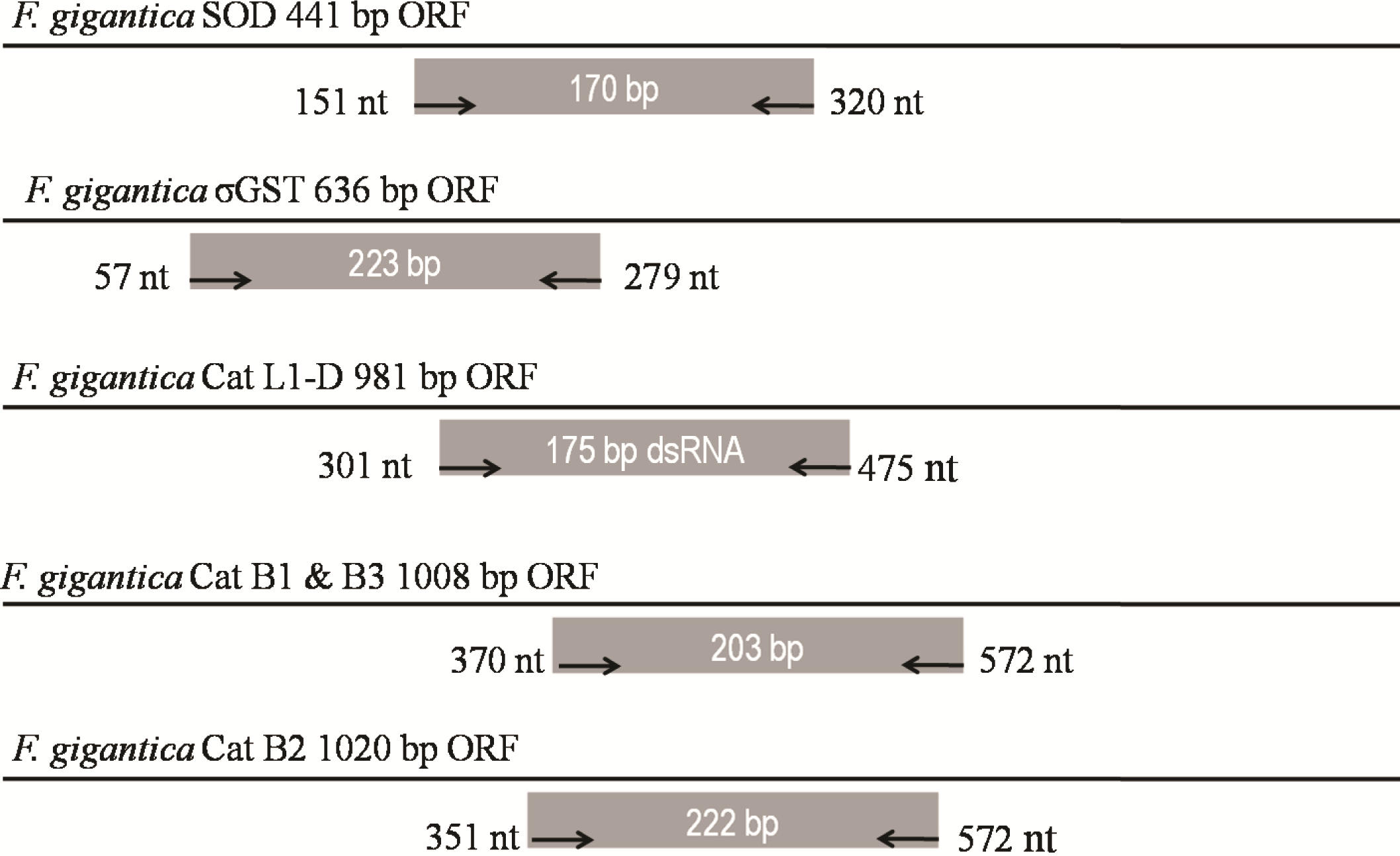

Supplement: S1 Fig — (TIF) [file pntd.0006109.s001.tif]
